# Establish a simple and quantitative deep learning-based method to analyse complicated intra- and inter-species social interaction behaviour for four stag beetle species

**DOI:** 10.1098/rsob.250060

**Published:** 2025-07-09

**Authors:** Michael Edbert Suryanto, Petrus Siregar, Tzong-Rong Ger, Chung-Der Hsiao

**Affiliations:** ^1^ Chung Yuan Christian University, Taoyuan, Taiwan; ^2^ Department of Chemistry, Chung Yuan Christian University, Taoyuan, Taiwan; ^3^ Department of Bioscience Technology, Chung Yuan Christian University, Taoyuan, Taiwan; ^4^ Department of Biomedical Engineering, National Yang Ming Chiao Tung University, Hsinchu, Taiwan; ^5^ Department of Biomedical Engineering, Chung Yuan Christian University, Taoyuan, Taiwan; ^6^ Department of Bioscience Technology, Chung Yuan Christian University, Taoyuan, Taiwan; ^7^ Department of Chemistry, Chung Yuan Christian University, Taoyuan, Taiwan

**Keywords:** stag beetle, social behaviour, deep learning, DeepLabCut, behavioural analysis, ethology

## Abstract

Stag beetles (Lucanidae) exhibit diverse social behaviours, yet quantifying these interactions remains challenging. Understanding social interactions within and between species is crucial for comprehending their behaviour, ecology and evolution. Stag beetles exhibit diverse social behaviours, including intraspecific competition, courtship and interspecific interactions, often involving complex physical displays and subtle cues. Traditional ethological methods for analysing these behaviours are time-consuming, subjective and limited in their ability to capture the nuances of dynamic interactions. This project aims to develop a simple and quantitative deep learning-based method to analyse complicated intra- and inter-species social interaction behaviour in four stag beetle species. This study utilizes DeepLabCut™ (DLC), a state-of-the-art deep learning-based pose estimation tool, to analyse and compare intra- and inter-species social interactions in four stag beetle species: *Phalacrognathus muelleri*, *Prosopocoilus astacoides*, *Dorcus titanus* and *Prosopocoilus inclinatus*. High-resolution videos of staged encounters were collected, and DLC was trained to accurately track key body parts of individual beetles. Behavioural parameters such as distance between individuals, orientation angles and movement trajectories were extracted from the pose data. Statistical analyses were conducted to identify species-specific differences in social behaviour, including aggression levels, courtship displays and dominance hierarchies. This study demonstrates the effectiveness of DLC in objectively quantifying complex social interactions in insects, providing valuable insights into the social ecology and evolutionary divergence of stag beetles.

**Figure:**
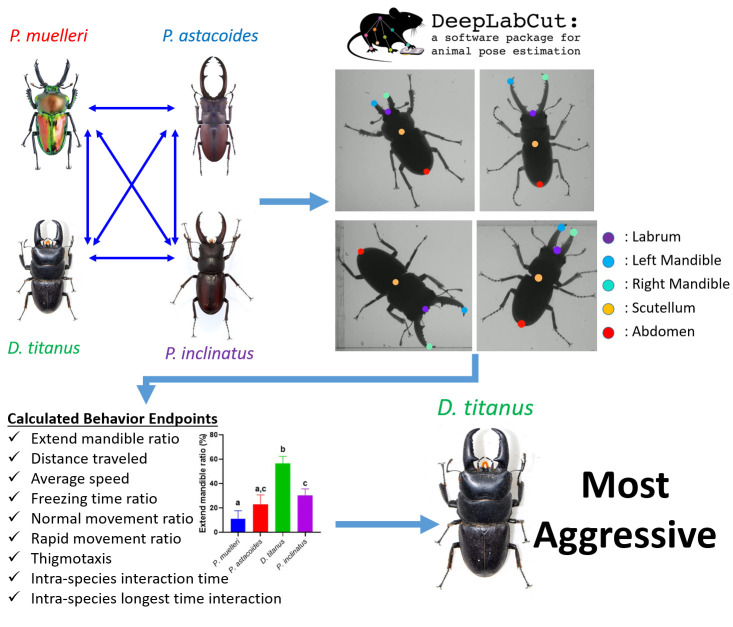

## 1. Introduction

Insects have various weapons to compete [1]. One group of insects with an extraordinary variety of weapons is the stag beetle family (Lucanidae). Stag beetles, characterized by their distinctive enlarged mandibles, are fascinating insects that exhibit a range of complex social behaviours. Understanding these behaviours is crucial for comprehending their ecology, evolution and conservation [2]. Stag beetle males fight for sap sites, territory, and females with their exaggerated mandibles [3]. The mandible and body sizes of male stag beetles can serve as reliable indicators of a species’ resource-holding capacity and are positively correlated with winning abilities [4]. Within and across species, stag beetles have a wide range of mandible sizes and shapes which might account for the highly variable behaviour they display in fighting contests [5]. While traditional ethological methods have been used to study insect behaviour [6], recent advancements in computer vision and deep learning have opened new avenues for more objective and quantitative analysis [7].

DeepLabCut™ (DLC), a state-of-the-art tool for markerless pose estimation, offers a powerful approach to tracking and analysing animal behaviour [8]. By utilizing deep neural networks, DLC can accurately identify and track key body parts of individuals within a video sequence, providing precise data on their movements and interactions [9]. New technologies like computer vision can enhance behavioural research on insects. This involves image capture, data extraction and analysis to quantify behavioural phenomena [10]. Insects are widely used as models in behavioural ecology due to their diverse behaviours and adaptability. Understanding their behaviour in natural contexts is crucial for studying the impact of environmental stressors [11]. However, most of the studies still depended on traditional methods, such as direct observation by observing insects in their natural habitat or in controlled environments [12] and ethograms with detailed catalogues of behaviours observed in a species [13]. Traditional methods have limitations: time-consuming, tedious, observer bias, consciously or unconsciously affecting the accuracy of data, and lack of advanced technology can limit the ability to measure and analyse complex behaviours [14–16]. These limitations have led to the development of modern techniques, such as computer vision and deep learning approach to complement and enhance traditional methods.

In this study, we propose to employ DLC to analyse and compare the intra- and inter-species social interaction behaviours of four stag beetle species: *Phalacrognathus muelleri*, *Prosopocoilus astacoides*, *Dorcus titanus* and *Prosopocoilus inclinatus*. Our research objectives are to develop a DLC model capable of accurately tracking and labelling key body parts in different stag beetle species. We employ a simple set-up recording video footage of intra- and inter-species interactions under controlled laboratory conditions. Then, we apply the DLC model to the recorded videos to extract quantitative data on behavioural parameters, including body posture, movement trajectories and interaction durations. By focusing on specific behavioural endpoints, such as mandible contact, chasing and mandible extension ratio, we aim to provide a robust and efficient framework for studying these interactions. Unlike traditional methods, our approach will streamline the analysis process, enabling researchers to obtain high-resolution behavioural data within a short time frame. Notably, our focus will not extend to long-term observations or determining outcomes such as winners or losers in competitive encounters. Instead, we aim to highlight key interaction behaviours that serve as reliable indicators of social dynamics. By doing so, we hope to overcome the limitations of traditional methods and contribute to a deeper understanding of stag beetle behaviour. Ultimately, this study will pave the way for more accessible and scalable analyses of complex social interactions in stag beetles and other species.

## 2. Methodology

### 2.1. Animal collection and husbandry

All the samples of *P. muelleri*, *P. astacoides*, *D. titanus* and *P. inclinatus* were obtained from a local insect store (Taipei, Taiwan). We used the male adults of stag beetle (eight males for each species). They were artificially reared under constant temperature: 20−22°C. Stag beetles were reared individually in separate transparent plastic pudding boxes containing decaying wood sprayed with water (50% water content). They were reared on commercial beetle jelly and their feed was regularly replaced [17]. A photoperiod of 15 h light and 9 h dark was set. They were acclimated to laboratory conditions for at least two weeks.

### 2.2. Behavioural observations

**Figure 1.**
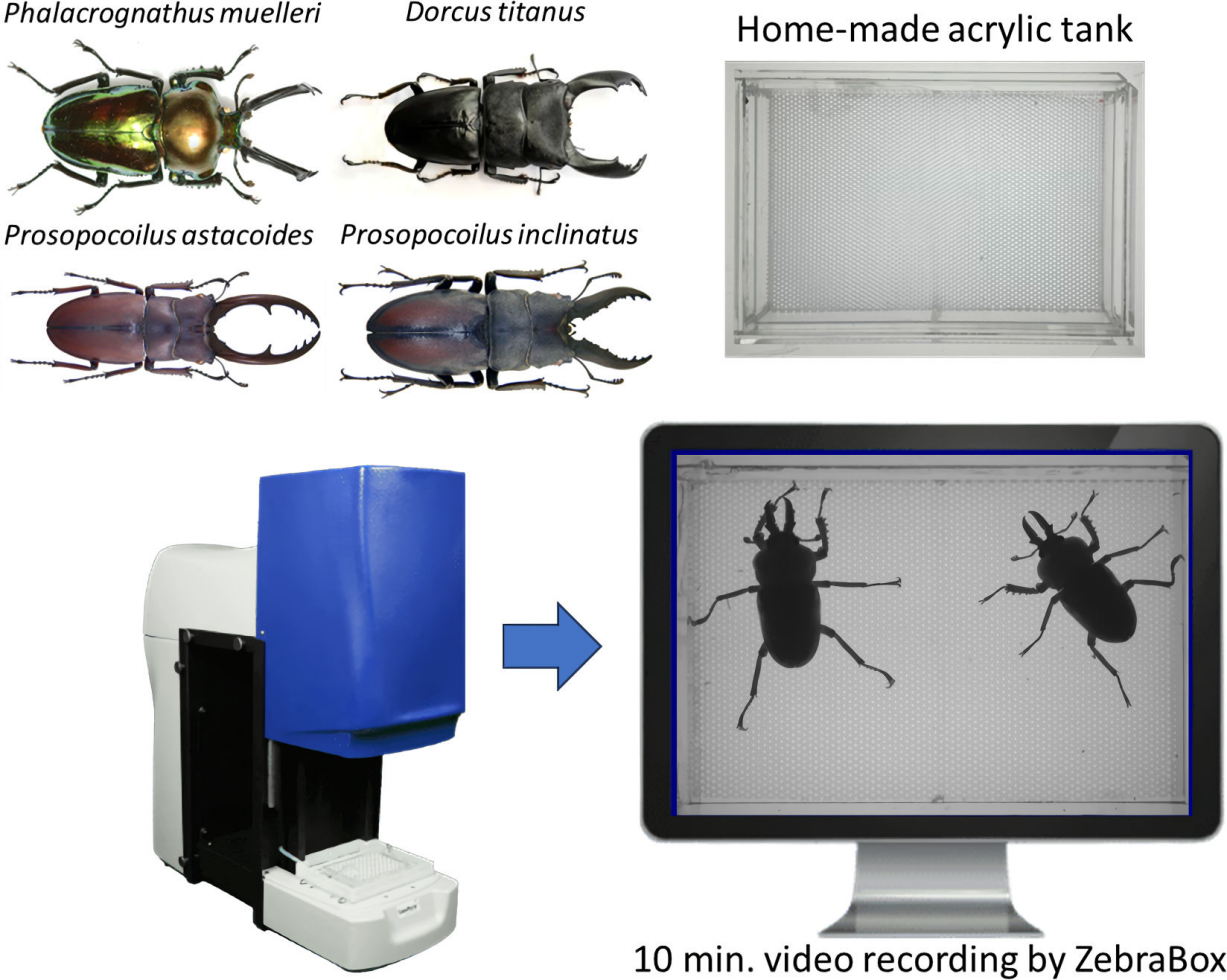
Experimental design and camera set-up setting for video recording. Four different stag beetle species were selected for this study: *P. muelleri, P. astacoides, D. titanus* and *P. inclinatus*. Two stag beetles were placed in a home-made acrylic tank and put into the ZebraBox. The video was recorded for 10 minutes and saved into .avi format.

The video recording was done using a ZebraBox viewpoint machine (ViewPoint Life Sciences, Lyon, France) with the light intensity set at the maximum level [18]. Two stag beetles were moved from the pudding box into a self-made acrylic plate of 13 × 8 cm^2^ (test plate). Given the small size of the test plate, stag beetles are likely to come in contact frequently to increase chances of aggressive motivation. The recording was conducted for 10 minutes. After the behaviour recording process, output videos were saved into .avi format with resolution 1024 × 768 and used in the following experiments (figure 1).

### 2.3. DeepLabCut model development

**Figure 2.**
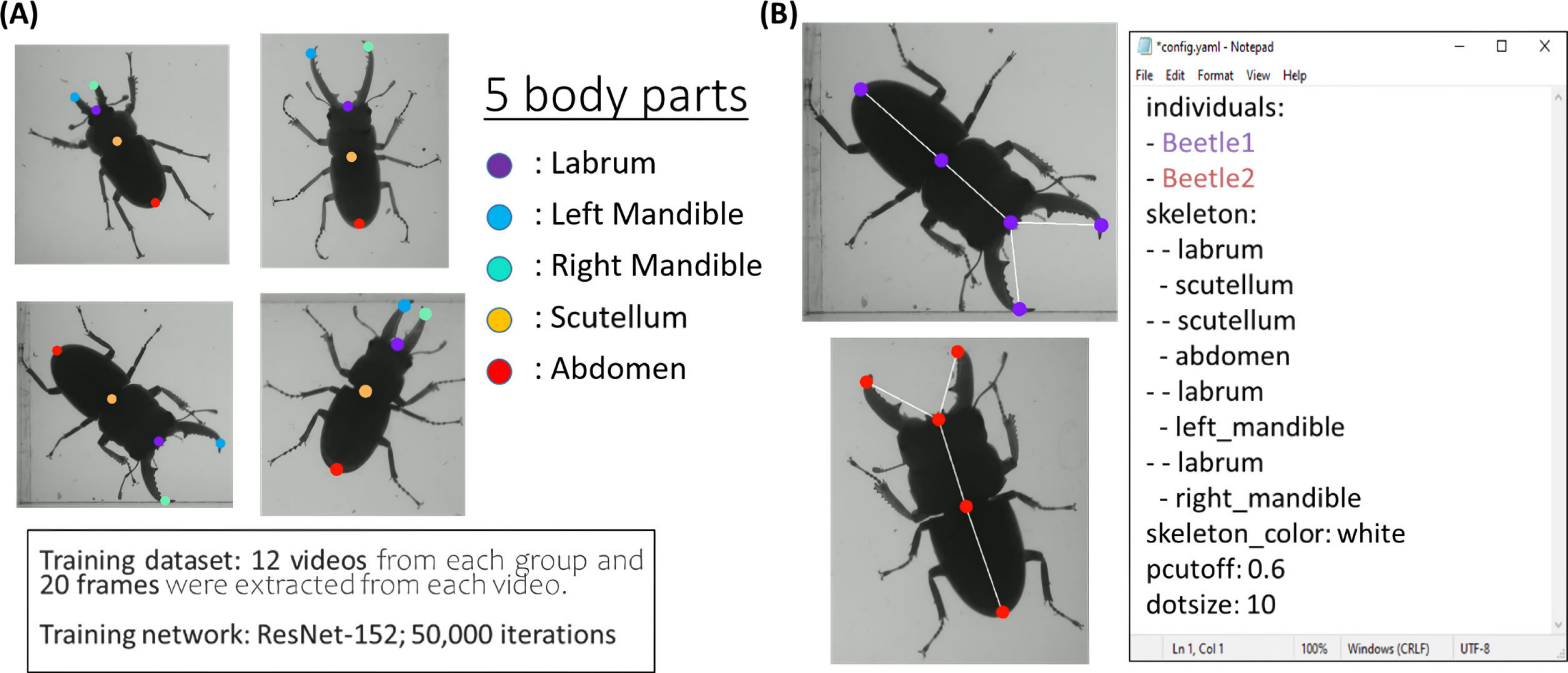
The training configuration for plot-poses estimation of multiple stag beetles generated by the DeepLabCut trained with ResNet-152 network. (A) Five body parts were labelled: labrum, left mandible, right mandible, scutellum and abdomen. (B) The configuration for skeleton plot was generated with p-cutoff: 0.6, size: 10, and colour: white.

The recorded videos are then used for the training process in DLC v.3.0, a markerless pose estimation tool to define body parts using deep learning [8]. We labelled five body parts of stag beetle: (i) labrum, (ii) left mandible, (iii) right mandible, (iv) scutellum and (v) abdomen (figure 2A). The result of labelled beetle’s body parts was marked by a circle dot with different colours. We trained the multiple beetle’s body parts configuration starting with 10 000 iterations by using ResNet-152 convolutional network. The initial step began with selecting 12 random videos, then extracting 20 frames automatically from each video using OpenCV’s K-means algorithm. Then, the training iterations were followed by increased intervals to 20 000; 30 000; 40 000; then finally until reaching a total of 50 000 iterations (electronic supplementary material, figure S1). Within the increased iterations, we also added eight new videos to be analysed then we refined the label position. The training was done with a total of 45 recorded videos (900 frames). To represent the exoskeleton of a stag beetle, a white line was drawn between labrum–scutellum, scutellum–abdomen, labrum–left mandible and labrum–right mandible (figure 2B). The network was evaluated after the training and was used for video analysis. The training cycles with the ResNet-152-based neural network [19] were used with default parameters. We validated with a single shuffle number, which yielded the test and train error (*X* and *Y* pixels). The *X* and *Y* coordinates were then conditioned for future analysis using a p-cutoff of 0.6 [20]. Furthermore, the output video was generated with animal identity coloured to differentiate between individuals.

### 2.4. Video analysis

The trained configurational network was used for analysis of a new batch of different videos. In this study, the data were divided into two groups: intra-species and inter-species. For intra-species, two stag beetles of the same species were put together in the test plate. Meanwhile, for the inter-species, two stag beetles of different species were put together into the test plate. By using the analysis feature in the DLC, we can retrieve *XY* coordinates from the labelled body parts. These coordinates were stored as .csv files which can be opened in Microsoft Excel for further analysis.

### 2.5. Behavioural parameter extraction

**Figure 3.**
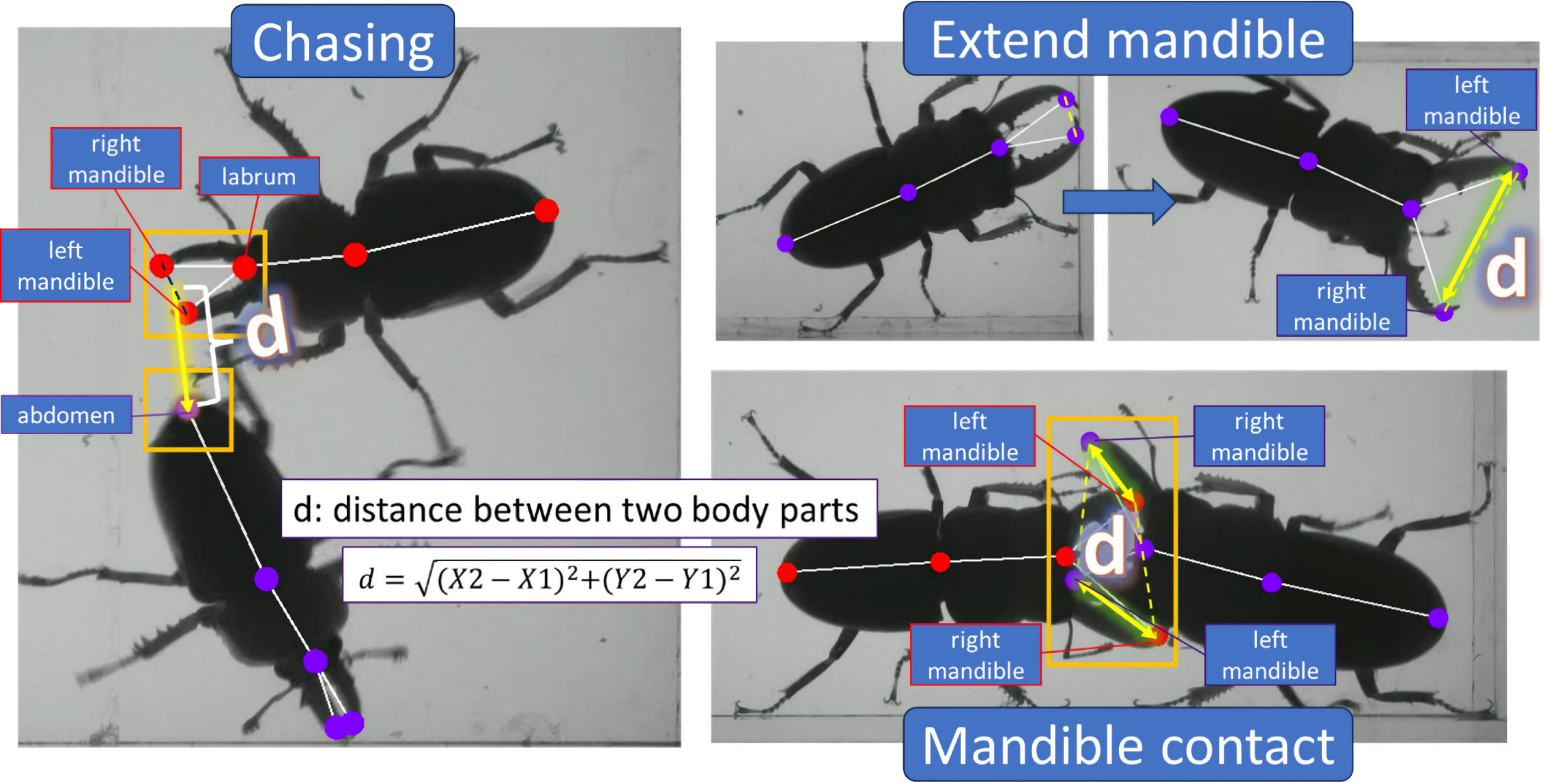
The outlier display of aggressiveness behaviour in stag beetles. Stag beetles chased their opponent, extended their mandible and have frequent mandible contacts. The quantitative way of measuring these behaviour endpoints was done by calculating the distance between two specific body parts.

We calculate key behavioural parameters, such as distance between individuals, orientation angles and movement speed. Several representative images of stag beetle’s behaviour have been evaluated in this study: the chasing, extended mandible and mandible contacts (figure 3). Stag beetles display aggressive or defensive behaviour by extending their mandible when threatened by their opponent. Moreover, they showed agonistic behaviour when they braced themselves against their opponents. In this scenario, one would have a winning experience and emerge as the dominant individual who usually chases off their opponent. All these endpoints were quantified using the distance between each specific body part (marked by a yellow line). Other behaviour endpoints, such as temporal patterns of interaction, including the frequency and duration of specific behaviours were also analysed. To measure these behaviour endpoints, we calculated the distance between two specific body parts. For example, in the extend mandible ratio, we calculated the distance between left and right mandible. Meanwhile, for chasing endpoint, we calculated the proximity distance between labrum and abdomen of two different individuals. Similarly, we applied the same quantitative way for the mandible contact ratio where the proximity distance of each mandible of two different individuals was calculated.

### 2.6. Statistical analysis

We use statistical tests to compare the mean values of behavioural parameters for intra-species and inter-species. All statistical analyses and graph plotting were conducted with GraphPad Prism (GraphPad Software v. 8 Inc., La Jolla, CA, USA). Initially, normality tests were conducted before the statistical analyses to evaluate the data distribution normality. Later, a Kruskal–Wallis test followed with Dunnet’s multiple comparisons test was used to compare the stag beetle groups, and all the data are expressed as mean with standard error of the mean (s.e.m.). The *p*-value was indicated by different letters a and b with significant differences *p* < 0.05. We also conducted correlation analyses to investigate the relationship between behavioural traits and morphological characteristics, such as body length and mandible length. We used DisplayR programming to visualize the Pearson correlation values between each trait [21].

## 3. Results

**Figure 4.**
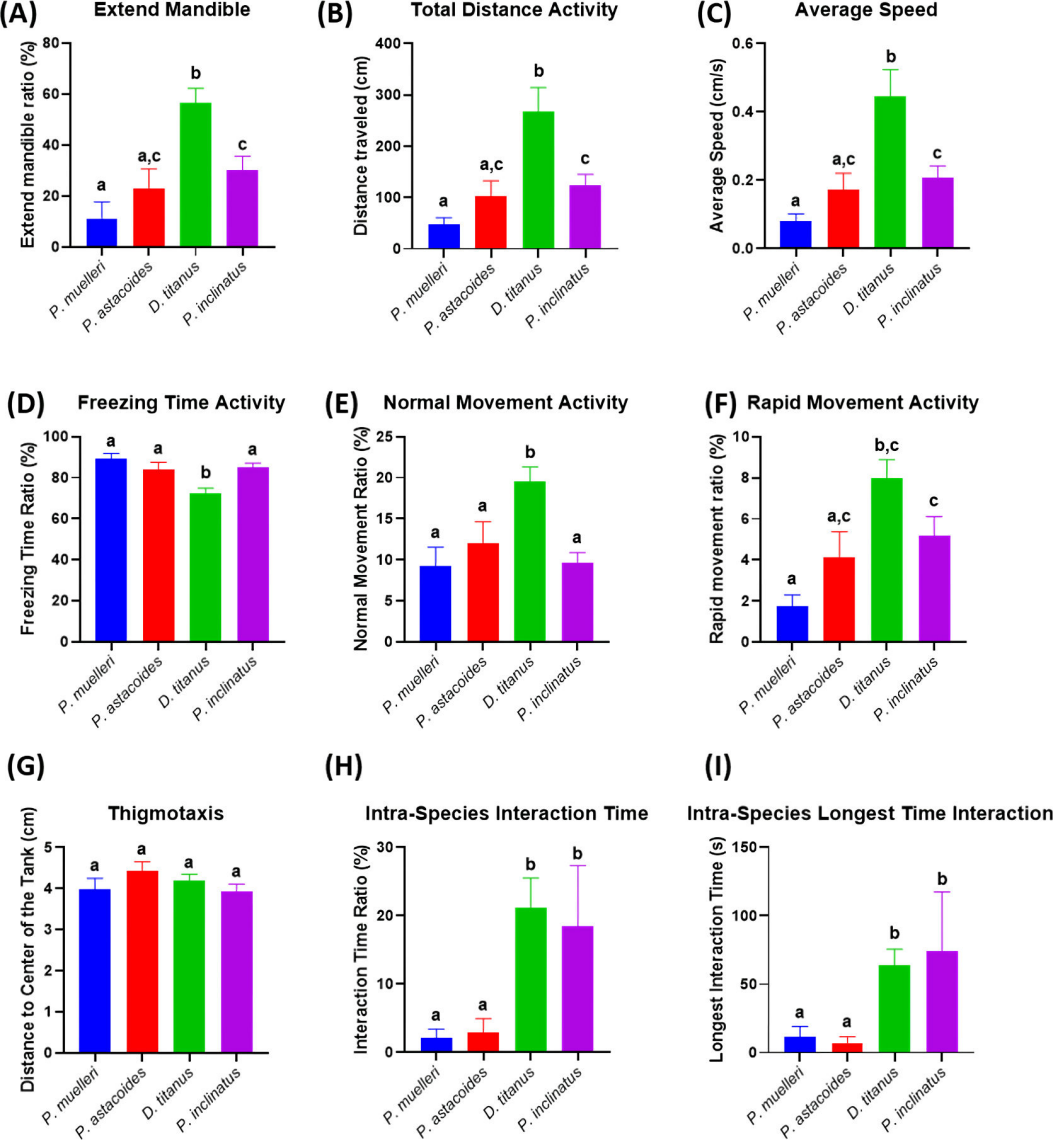
Comparison of locomotor activity between intra-species interaction of four different stag beetle species for 10  minutes. Nine locomotor endpoints of (A) mandible extended percentage, (B) total distance, (C) average speed, (D) freezing time, (E) normal movement, (F) rapid movement, (G) thigmotaxis, (H) inter-beetle interaction time and (I) longest time interaction were statistically analysed by Kruskal–Wallis with Dunn’s multiple comparisons test. Different letters indicate statistically significant differences (*p* ≤ 0.05). Mean ± s.e.m. is used to express the data (*n* = 14).

This study is expected to provide valuable insights into the social behaviour of stag beetles. By quantifying and comparing the behaviour of different species, we aim to identify species-specific behavioural strategies by discovering unique adaptations that allow each species to thrive in its specific ecological niche. To understand the role of social interactions by exploring how social behaviour influences survivability, resource acquisition and predator avoidance. We also can gain insights into the evolution of social behaviour by investigating the evolutionary history of social behaviour in stag beetles and identify potential selective pressures. Based on the defensive behaviour mechanism towards threat displays, stag beetles may raise their mandibles and open them wide to appear more intimidating. This can deter predators or rivals. Based on the results, *P. muelleri* has the lowest extended mandible ratio among the groups (figure 4A). Kruskal–Wallis test identified significant differences in the mandible extension ratio across species (*p* < 0.05). Specifically, *P. muelleri* exhibited the lowest extended mandible ratio compared to the other groups (suggesting reduced aggressive or defensive displays relative to other species). *D. titanus* has the highest total distance and average speed (figure 4B,C). Compared to other species, *D. titanus* is more active and robust. Pairwise comparisons revealed that *D. titanus* had the highest total distance travelled and average speed compared to all other species (*p* < 0.05, Dunn’s test). This result potentially reflected high levels of exploratory or territorial behaviour. Additionally, *D. titanus* exhibited significantly less freezing time (*p* < 0.05) and a higher proportion of rapid movement (*p* < 0.05) than the other species, indicating its higher responsiveness and dynamic movement patterns. *D. titanus* and *P. inclinatus* might display different behaviours, such as variations in aggression, territoriality or social interactions. Both species have significant spikes in social interaction compared to *P. muelleri* and *P. astacoides* (figure 4H,I). The significant differences in behaviour compared to other species indicate unique ecological adaptations. The variability in activity levels, mandible extension ratio and social interaction behaviours suggests niche differentiation and specialized roles within their respective environments. Meanwhile for other endpoints, the thigmotaxis showed no significant difference among these four species (figure 4G).

**Figure 5.**
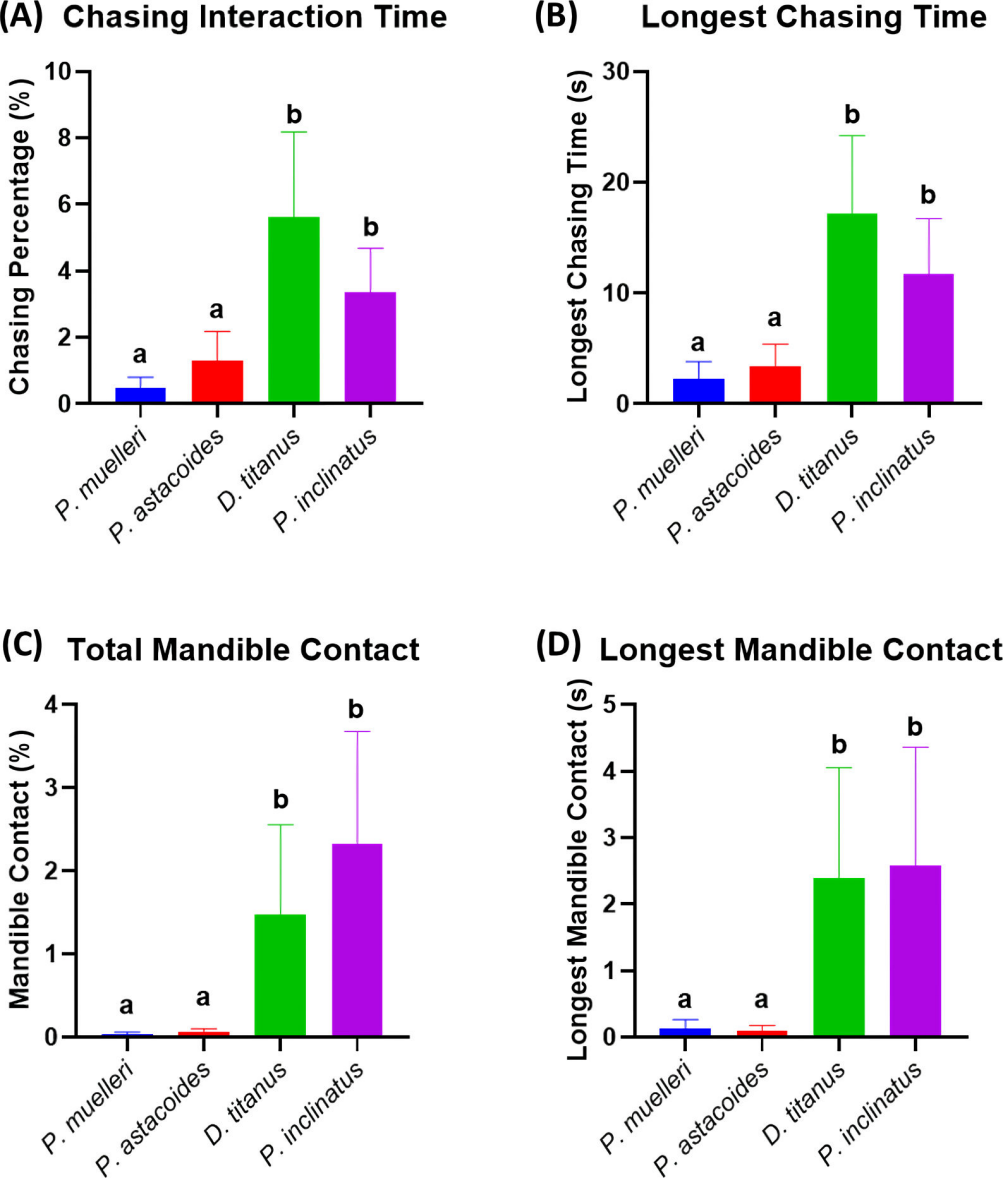
Comparison of agonistic behaviour in intra-species of stag beetles. Chasing interaction endpoints: (A) total chasing interaction time (%) and (B) longest chasing time (s). Two fighting endpoints were measured: (C) total mandible contact (%) and (D) the longest mandible contact (s). The graphs are plotted as columns with mean ± s.e.m. The statistical differences were analysed by the Kruskal–Wallis test (*n* = 14 for each group; different letters a and b indicate significant differences between variables with *p* < 0.05).

The analysis of chasing and mandible contact behaviours revealed significant inter-species differences, particularly highlighting the aggressive tendencies of *D. titanus* and *P. inclinatus*. In the chasing behaviour, Kruskal–Wallis test identified significant differences in chasing behaviour among the four species (*p* < 0.05). *D. titanus* and *P. inclinatus* displayed significantly higher chasing ratio compared to *P. muelleri* and *P. astacoides* (figure 5A,B). This elevated chasing activity suggests a more active form of territorial aggression or dominance display in these two species, consistent with previous findings on the role of chasing in establishing dominance hierarchies in stag beetles. Meanwhile, for mandible contact (fighting) behaviour also supporting the result. Similar to chasing, significant differences were observed in the mandible contact ratio (*p* < 0.05, Kruskal–Wallis test). *D. titanus* and *P. inclinatus* exhibited significantly higher mandible contact ratios compared to the other species (figure 5C,D).

**Figure 6.**
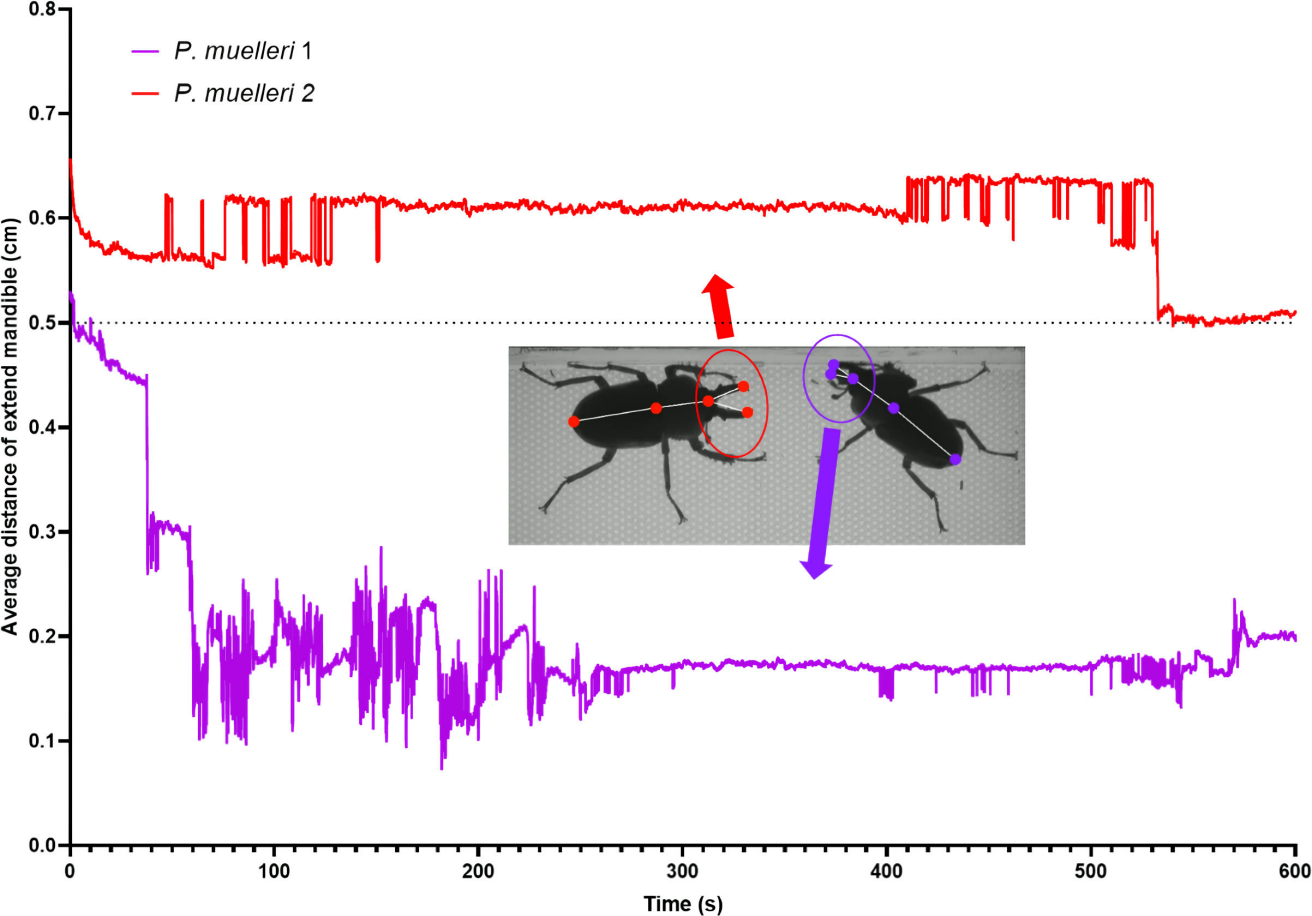
Comparison of average extend mandible distance in *P. muelleri* group. The graph is plotted as an *XY* line with a time maximum of 600 s. The representative image of extended mandible was displayed from *P. muelleri* 2, while closed mandible position was displayed by *P. muelleri* 1.

The representative image presented in figure 6 demonstrates the average mandible extension distances in the *P. muelleri* group over a 600-second observation period. The threshold for mandible extension was set at 0.5 cm, with any recorded mandible distance exceeding this value classified as an extended mandible. The red plot represents the mandible distance of *P. muelleri* 2, which consistently maintained a mandible extension above the 0.5 cm threshold during the initial phase of the observation. A significant drop below the threshold was observed midway through the trial, indicating a shift to a closed mandible posture. The purple plot, corresponding to *P. muelleri* 1, predominantly remained below the 0.5 cm threshold, displaying minimal mandible extension throughout the experiment. The representative images included in the figure visually depict the contrasting mandible positions of the two individuals. *P. muelleri* 2 illustrates an extended mandible posture, while *P. muelleri* 1 demonstrates a closed mandible posture. Overall, the results suggest distinct behavioural patterns between individuals in the group. *P. muelleri* 2 exhibited more frequent and sustained mandible extension compared to *P. muelleri* 1, which may indicate a higher level of aggressive or defensive behaviour.

**Figure 7.**
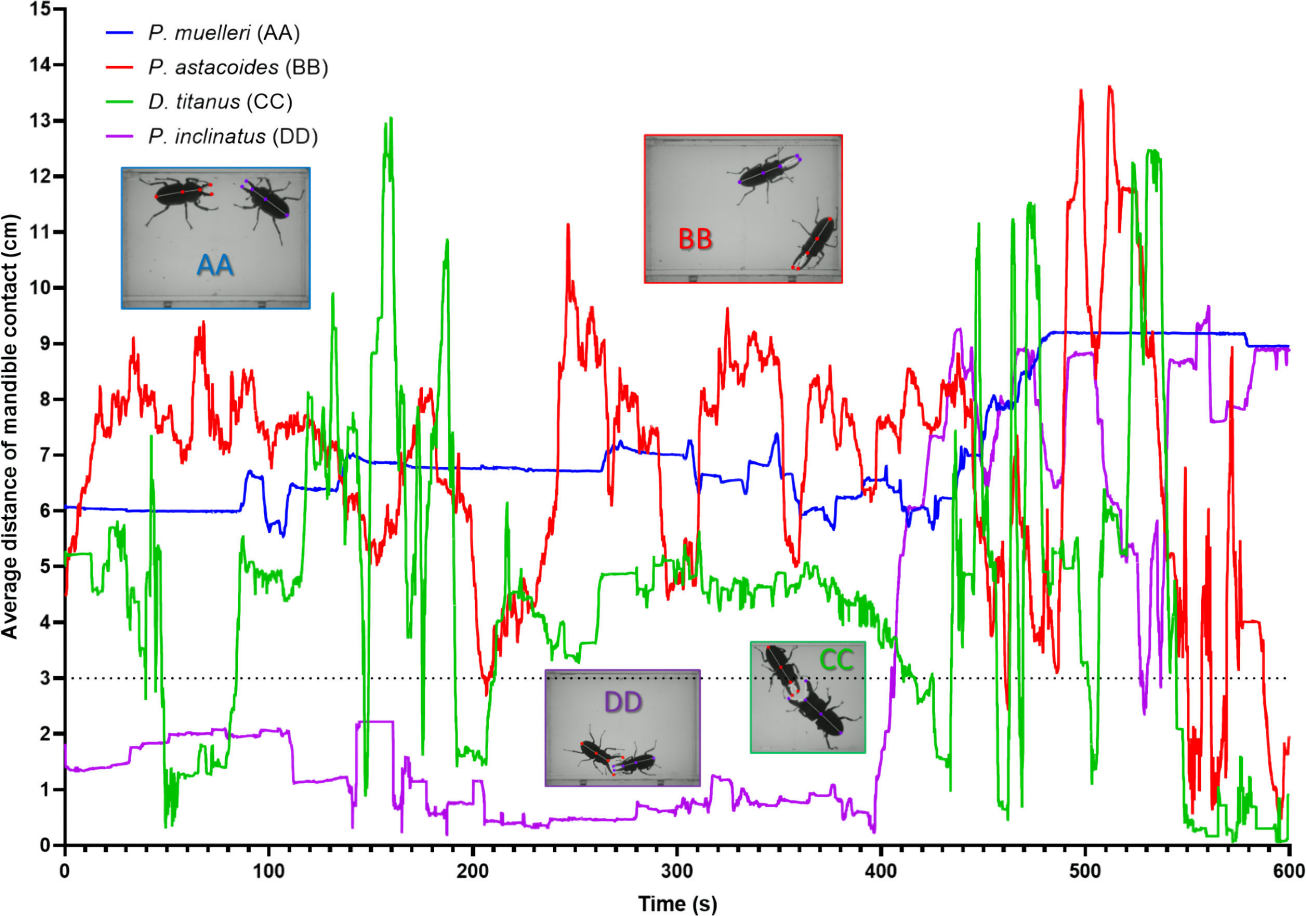
Comparison of average distance of mandible contact in four different stag beetle species: *P. muelleri* (AA), *P. astacoides* (BB), *D. titanus* (CC) and *P. inclinatus* (DD). The graph is plotted as an *XY* line with a time maximum of 600 s.

The temporal dynamics of mandible contact distances among four stag beetle species, *P. muelleri* (AA), *P. astacoides* (BB), *D. titanus* (CC) and *P. inclinatus* (DD), are illustrated in figure 7. Threshold was set at 3 cm. Distance position between two stag beetles below 3 cm will count as mandible contact. Over the 600-second observation period, the species demonstrated distinct behavioural trends. In group AA, the mandible contact distance remained consistently high throughout the trial (mean ± SD: 7.111 ± 1.143), with occasional minor peaks. This indicates minimal engagement or a reserved aggression pattern. Similarly in group BB, a significant increase in distance of mandible contact was observed, peaking frequently (mean ± SD: 6.825 ± 2.248). This pattern suggests a low level of aggressive interactions and temporary mandible engagement. Meanwhile, in group CC, the contact distance for this species exhibited a gradual rise with consistent oscillations (mean ± SD: 4.482 ± 2.589), suggesting moderate aggression with controlled and periodic mandible usage. Group DD also displayed aggression with the mandible contact distance remaining mostly stable at a tight range (mean ± SD: 3.168 ± 3.126), with minimal fluctuations, reflecting high aggression or fighting behaviour. The graph highlights clear interspecific differences in mandible usage during interactions. *D. titanus* and *P. inclinatus* displayed the most pronounced and sustained increases in mandible contact distance, suggesting it is the most aggressive species under the experimental conditions. Conversely, *P. muelleri* and *P. astacoides* demonstrated lower aggression levels, as indicated by their relatively stable and minimal contact distances. The trajectory movement pattern of intra-species from each group is summarized in electronic supplementary material, figure S2.

**Figure 8.**
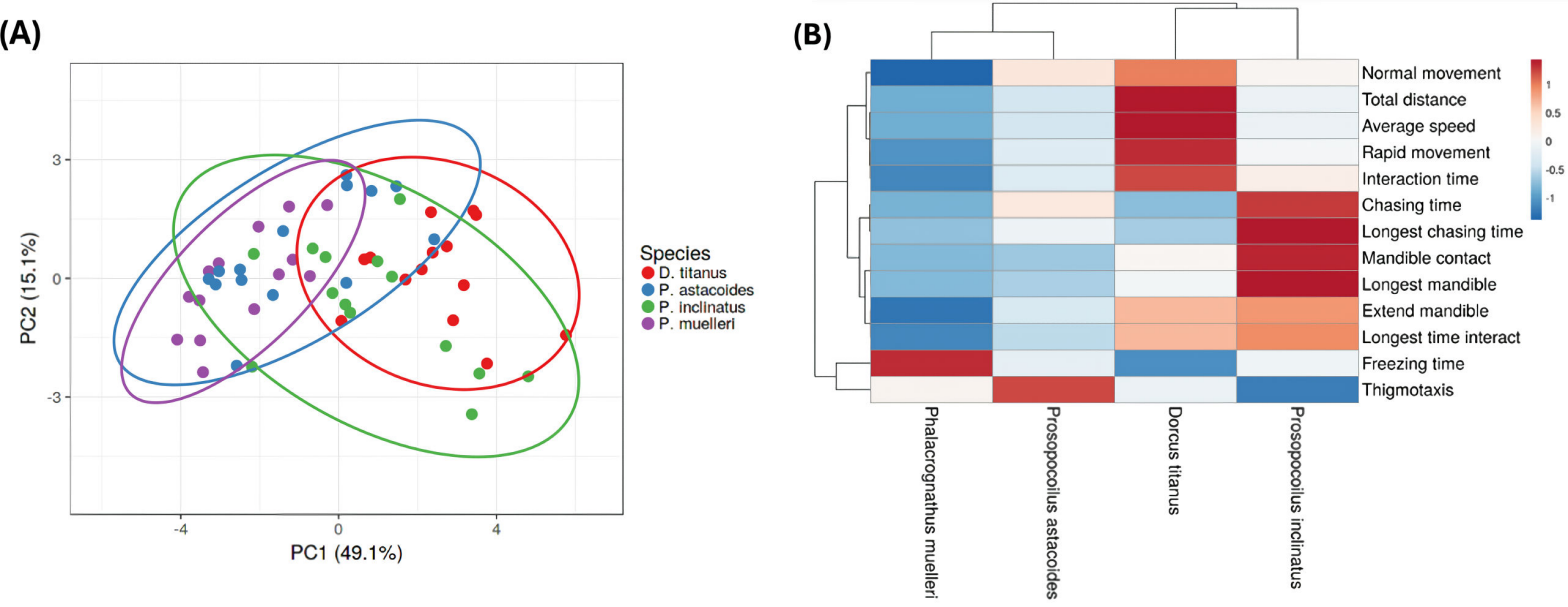
Principal component analysis (A) and heatmap clustering (B) of intra-species stag beetle behaviour. Four different species, *P. muelleri*, *P. astacoides*, *D. titanus* and *P. inclinatus*, were sub-divided into two major cluster groups. Blue circle: *P. muelleri* and *P. astacoides*; red circle: *D. titanus* and *P. inclinatus*.

To assess whether behavioural patterns cluster consistently within species, principal component analysis (PCA) was conducted on data from all individual pairs rather than pooling species-level data. Each point on the PCA plot represents an individual pair of beetles, with behavioural endpoints as input features. This approach ensures a more granular analysis of inter- and intra-species variability. The PCA results (figure 8) show distinct clustering of individuals within the same species, supporting the reliability of the analysis and capturing the primary behavioural differences. Consistent clustering of individuals within species reinforces the robustness of behavioural distinctions, while minimizing the influence of outliers. It revealed distinct clustering patterns among the four stag beetle species, indicating significant behavioural differences. As shown in figure 8A, three major clusters were identified based on behavioural traits. The first cluster (blue and purple circles) comprised *P. muelleri* and *P. astacoides*, which shared similar behavioural characteristics with low activity values in almost all behaviour endpoints tested. The second cluster (red circle) and third cluster (green circle) included *D. titanus* and *P. inclinatus*, reflecting a separate grouping with distinct behaviours. The first principal component (PC1) accounted for 49.1% of the variation, while the second principal component (PC2) explained 15.1%, cumulatively capturing 64.2% of the total variation in intra-species behavioural data. The heatmap clustering analysis (figure 8B) further elucidated the behavioural differences across species by grouping them based on specific metrics. The dendrogram visually highlights two major clusters consistent with the PCA results. Behavioural traits such as normal movement, total distance and average speed were less prominent in *P. muelleri* and *P. astacoides*, aligning with their inclusion in the blue cluster. Conversely, chasing time, mandible contact and longest mandible extension time were more pronounced in *D. titanus* and *P. inclinatus*, supporting their grouping in the red cluster. Interestingly, freezing time and thigmotaxis were observed with variable patterns across species, indicating species-specific responses to interaction dynamics. These findings highlight clear inter-species distinctions in behavioural repertoires and emphasize the utility of PCA and clustering methods for categorizing complex behavioural data.

**Figure 9.**
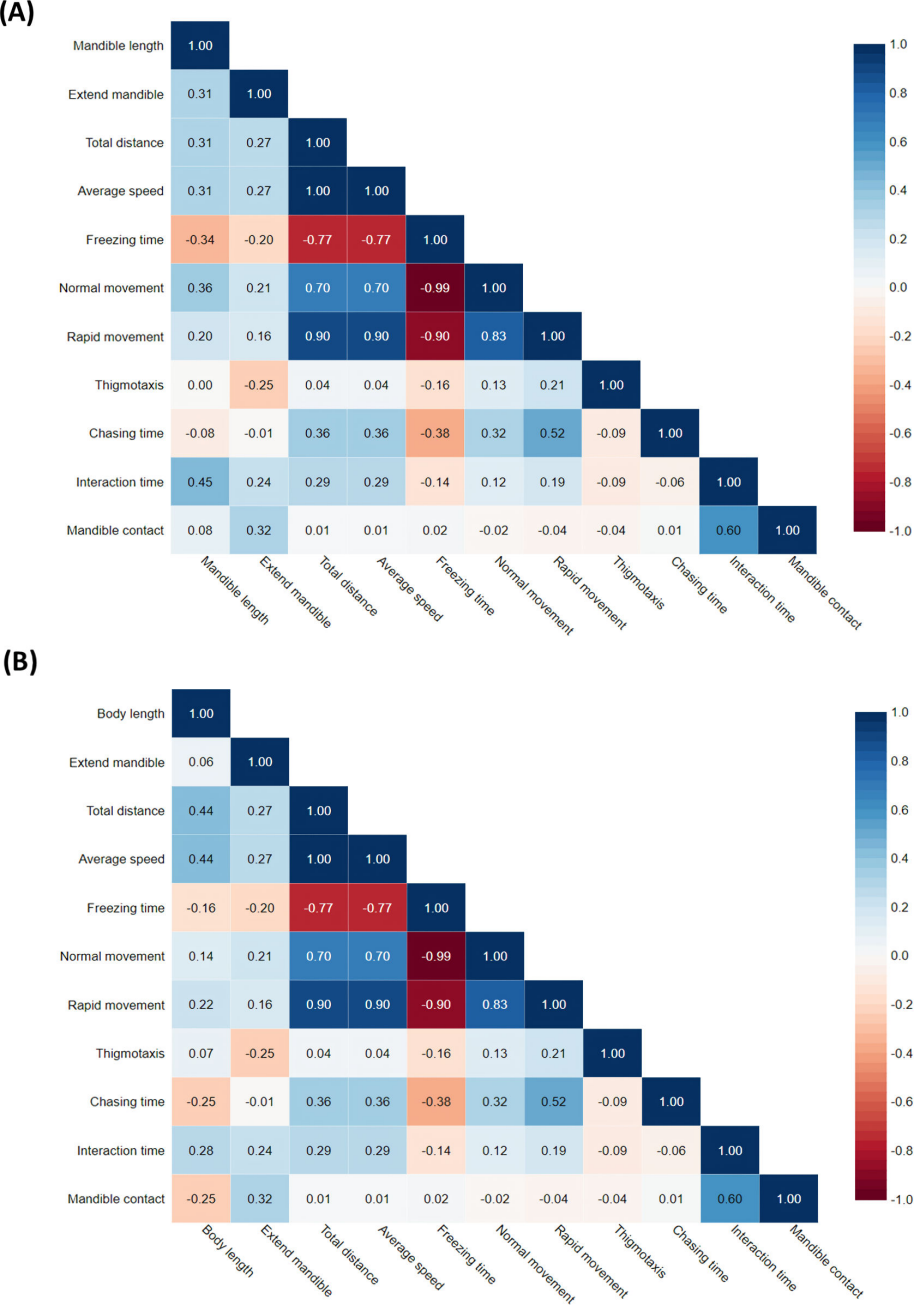
The visualization of Pearson correlation values between traits using R script. (A) A heatmap of correlation matrix between mandible length and behavioural endpoints tested. (B) A heatmap of correlation matrix between body length and behavioural endpoints tested.

The Pearson correlation coefficient (*r*) indicates the magnitude and direction of linear relationships between two variables. To interpret the values: +1 (perfect positive correlation: as one variable increases and the other also increases); −1 (perfect negative correlation: as one variable increases and the other decreases); and 0 (no correlation with no linear relationship between the variables). The Pearson correlation test was conducted to examine the relationship between insect mandible length, body length and aggressive behaviour endpoints. The analysis revealed a low correlation between mandible length and aggressive behaviour (*r* = 0.31), indicating a weak positive relationship (figure 9A). Similarly, the correlation between body length and aggressive behaviour (figure 9B) was also low (*r* = 0.06), suggesting that these morphological traits have a minimal impact on the aggressive behaviour of the insects studied. However, positive correlation was still found between total distance and body length (*r* = 0.44). These findings suggest that other factors, possibly environmental or genetic, may play a more significant role in influencing aggressive behaviour in insects. Further research is needed to identify and understand these contributing factors.

**Figure 10.**
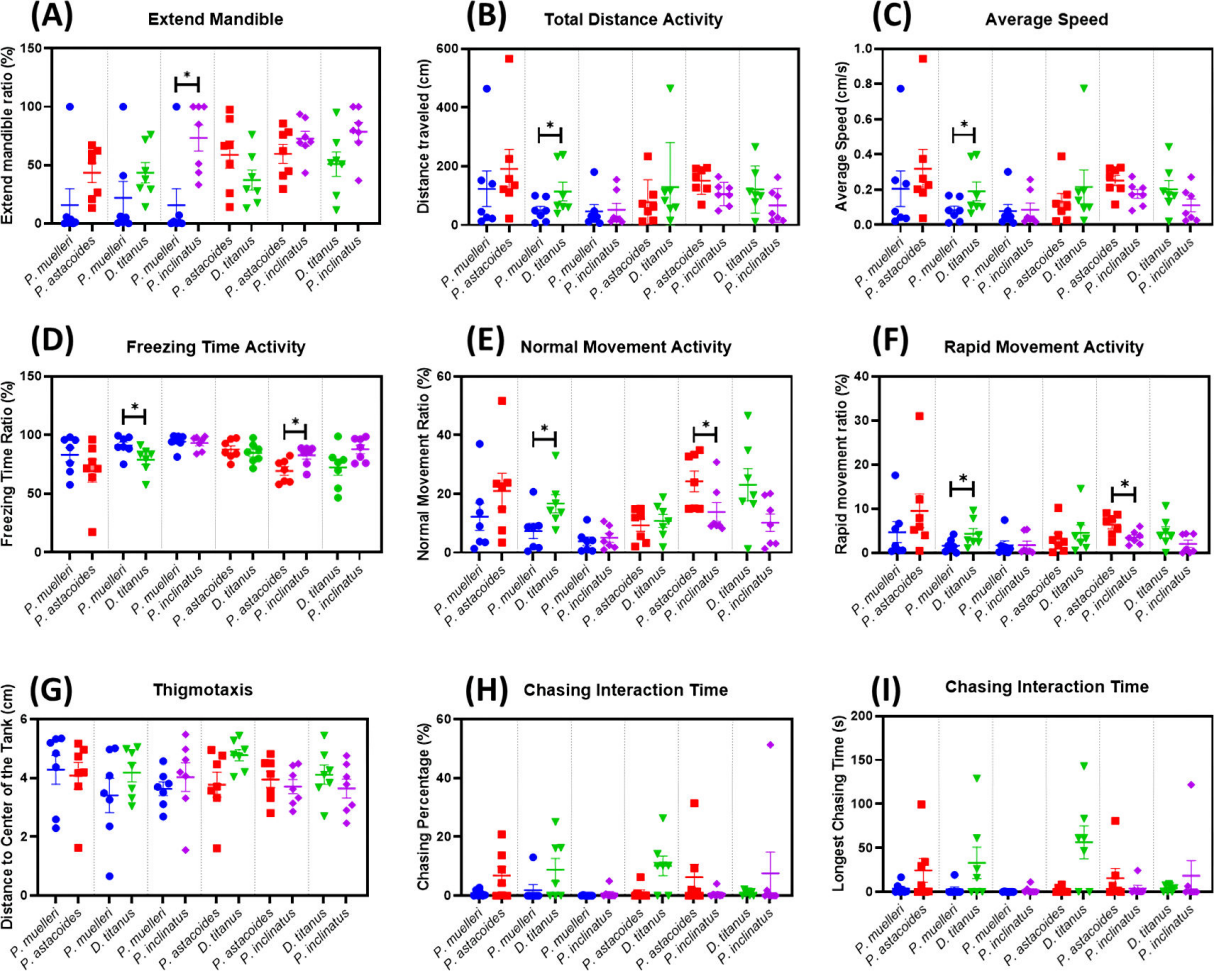
Comparison of locomotor activity between inter-species interaction of four different stag beetle species for 10  minutes. Total of six combination groups: *P. muelleri–P. astacoides*, *P. muelleri–D. titanus*, *P. muelleri–P. inclinatus*, *P. astacoides–D. titanus*, *P. astacoides–P. inclinatus* and *D. titanus–P. inclinatus*. Nine locomotor endpoints of (A) mandible extended percentage, (B) total distance, (C) average speed, (D) freezing time, (E) normal movement, (F) rapid movement, (G) thigmotaxis, (H) inter-beetle interaction time and (I) longest time interaction were statistically analysed by Kruskal–Wallis with Dunn’s multiple comparisons test. Different letters indicate statistically significant differences (*p* ≤ 0.05). Mean ± s.e.m. is used to express the data (*n* = 14).

The behavioural interactions between the inter-species stag beetle groups were evaluated under specific encounter scenarios using the Wilcoxon matched-pairs signed rank test (*p* < 0.05; number of pairs = 7). The findings are summarized as displayed in figure 10. In encounters between *P. inclinatus* and *P. muelleri*, *P. inclinatus* displayed a significantly higher extended mandible ratio compared to *P. muelleri* (figure 10A). This result suggests that *P. inclinatus* exhibits a more aggressive defensive posture in direct encounters. For other pairs group, *D. titanus* demonstrated significantly higher locomotor activity during interactions with *P. muelleri*, as shown by significant increase in total distance travelled and average speed (figure 10B,C). This included greater normal movement activity and significantly less freezing time activity (figure 10D,E). These behaviours highlight the active and dynamic response of *D. titanus* in competitive scenarios. In contrast, during encounters between *P. muelleri* and *D. titanus*, *P. muelleri* showed a significant reduction in rapid movement activity (figure 10F). This behaviour may indicate a strategy of reduced energy expenditure or a shift in defensive tactics when confronted with the more robust and active *D. titanus*. When encountering *P. astacoides*, *P. inclinatus* exhibited a significant reduction in rapid movement activity and an increase in freezing time activity (figure 10D–F). This suggests a behavioural adjustment, possibly reflecting a defensive or stress-induced response to the interaction with *P. astacoides*. These results emphasize the species-specific behavioural adaptations and dynamics during competitive interactions. The observed differences underline the potential ecological and evolutionary drivers influencing the locomotion, movement activity and aggressive behaviours of these stag beetles. The trajectory movement pattern of inter-species stag beetles from six combination groups is summarized in electronic supplementary material, figure S3.

**Figure 11.**
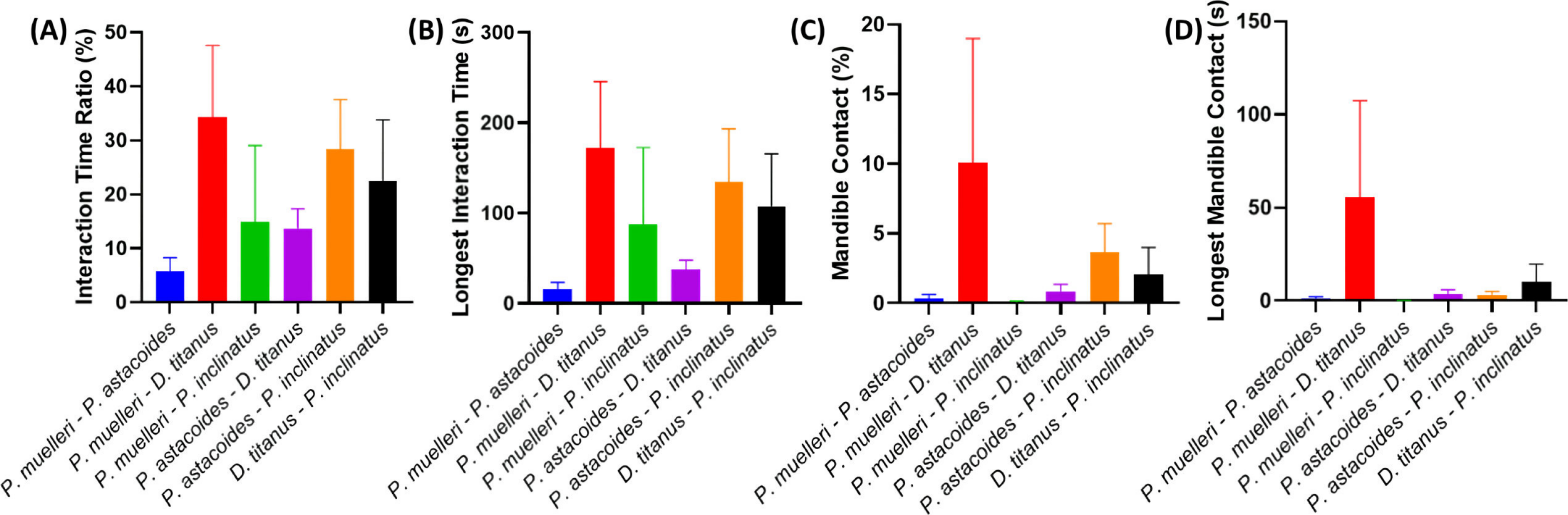
Comparison of social interaction and agonistic behaviour in inter-species of stag beetles. A total of six combination groups: *P. muelleri–P. astacoides*, *P. muelleri–D. titanus*, *P. muelleri–P. inclinatus*, *P. astacoides–D. titanus*, *P. astacoides–P. inclinatus* and *D. titanus–P. inclinatus*. Social interaction endpoints: (A) total interaction time ratio (%) and (B) longest interaction time (s). Agonistic behaviour endpoints were measured: (C) total mandible contact (%) and (D) the longest mandible contact (s). The graphs are plotted as columns with mean ± s.e.m. The statistical differences were analysed by Dunn’s multiple comparisons test (*n* = 14).

The results of the study on inter-species social interaction and agonistic behaviour in stag beetles are presented in figure 11. The bar graphs illustrate the mean values and s.e.m. for various endpoints across different conditions. The study included six combination groups of stag beetle species: *P. muelleri–P. astacoides* (AB), *P. muelleri–D. titanus* (AC), *P. muelleri–P. inclinatus* (AD), *P. astacoides–D. titanus* (BC), *P. astacoides–P. inclinatus* (BD) and *D. titanus–P. inclinatus* (CD). Based on the results, it is evident that group AB has the lowest interaction time ratio, while group AC has the highest interaction time ratio (figure 11A). Similar to the total interaction time ratio, group AB has the shortest interaction time, while group AB has the longest interaction time (figure 11B). The other four groups have intermediate values and fall in between. However, no significant differences were observed across these interaction endpoints. For the agonistic behaviour, group AC has the lowest mandible contact percentage, while group AB has the highest mandible contact (figure 11C). Group AD has the shortest mandible contact time, while group AB has the longest mandible contact time (figure 11D). The results suggest that each species exhibits distinct patterns of social interaction and agonistic behaviour compared to the other groups. However, statistical analysis using Dunn’s multiple comparisons test revealed no significant differences between the groups for all measured endpoints. Based on these results, it revealed that each combination group exhibited unique patterns of social interaction and agonistic behaviour. The results indicate that the interaction time and mandible contact varied significantly across different species combinations, highlighting the influence of species-specific behaviours on inter-species interactions.

**Figure 12.**
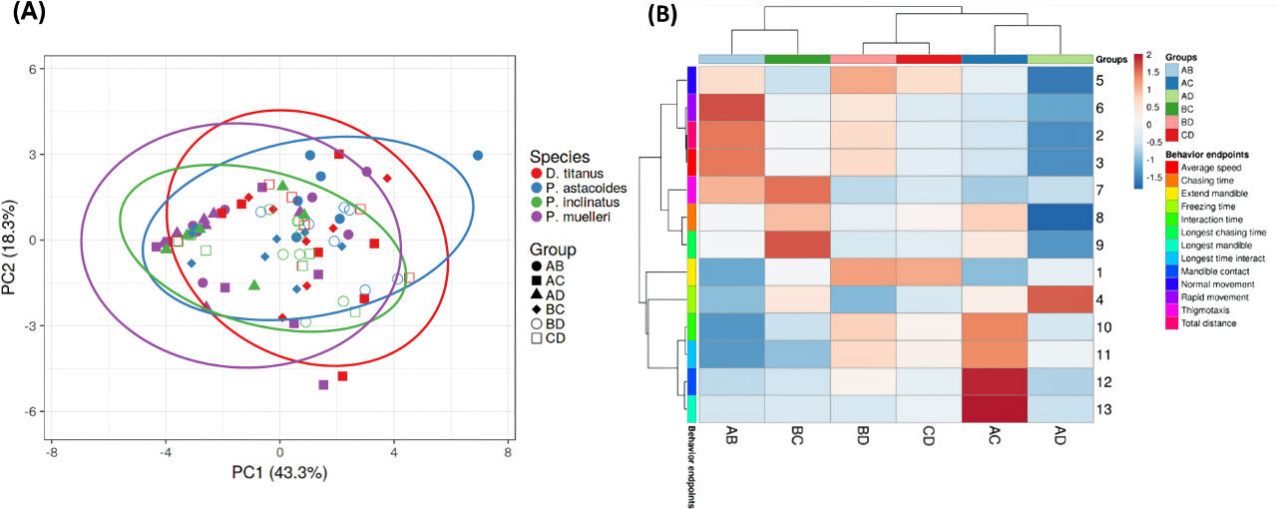
Principal component analysis (A) and heatmap clustering (B) of inter-species stag beetle behaviour. Total of six combination groups: *P. muelleri–P. astacoides* (AB), *P. muelleri–D. titanus* (AC), *P. muelleri–P. inclinatus* (AD), *P. astacoides–D. titanus* (BC), *P. astacoides–P. inclinatus* (BD) and *D. titanus–P. inclinatus* (CD).

Individual pair data were used instead of pooled data to ensure that observed patterns reflect true behavioural variability. The clustering algorithm was calibrated using Euclidean distance with average linkage, and robustness was assessed using silhouette scores and intra-cluster variance metrics. In figure 12A, the PCA plot displays clustering patterns that align with species-specific characteristics. Individual pairs of the same species consistently form tight clusters, which strengthens the claim of distinct inter-species behavioural patterns. PC1 explained 43.3% of the behavioural variance, while PC2 accounted for 18.3%, cumulatively capturing 61.6% of the total variation. Distinct clusters corresponding to species suggest that individuals of the same species exhibit similar behavioural patterns. For instance, *D. titanus* (red) forms a relatively tight cluster, reflecting consistent species-specific traits. In contrast, the proximity of *P. muelleri* (purple) to other species in PCA space indicates some overlap in behavioural endpoints, potentially reflecting shared ecological adaptations or experimental conditions. Furthermore, group-level clustering, such as those of beetle pairs (e.g. AB, BC, CD), highlights inter-species relationships. While some group proximities align well with hierarchical clustering (e.g. BC and BD), inconsistencies such as AC and CD belonging to different branches despite appearing proximately in PCA space are noted. These observations warrant further analysis of clustering metrics to better substantiate grouping patterns. Overall, the PCA results emphasize that species-specific behavioural strategies can be quantitatively assessed through observed endpoints. The clustering of individuals within the same species reinforces the reliability of the analysis, while deviations or overlaps provide opportunities to explore behavioural diversity and ecological adaptability further.

The heatmap clustering analysis (figure 12B) further highlighted differences in interaction behaviours across the inter-species groups. Group clustering aligned with the PCA results, reinforcing the proximity of groups AC and AD, as well as BD and CD. Groups AB and BC formed separate clusters, distinguishing their behavioural patterns. Behavioural endpoints such as average speed, chasing time and interaction time were highly variable across groups, serving as key factors for differentiation. Groups involving *P. inclinatus* (e.g. BD and CD) exhibited prominent behaviours such as mandible contact, extended mandible time and longest time interacting, suggesting more intensive interactions. In contrast, groups involving *P. muelleri* (e.g. AB, AC and AD) demonstrated higher levels of normal movement and total distance travelled, indicating less aggressive or more exploratory behaviours during interactions. Overall, the results highlight distinct behavioural profiles for each inter-species group, with notable similarities between closely related clusters. This analysis provides a comprehensive view of inter-species interaction dynamics among stag beetles.

**Figure 13.**
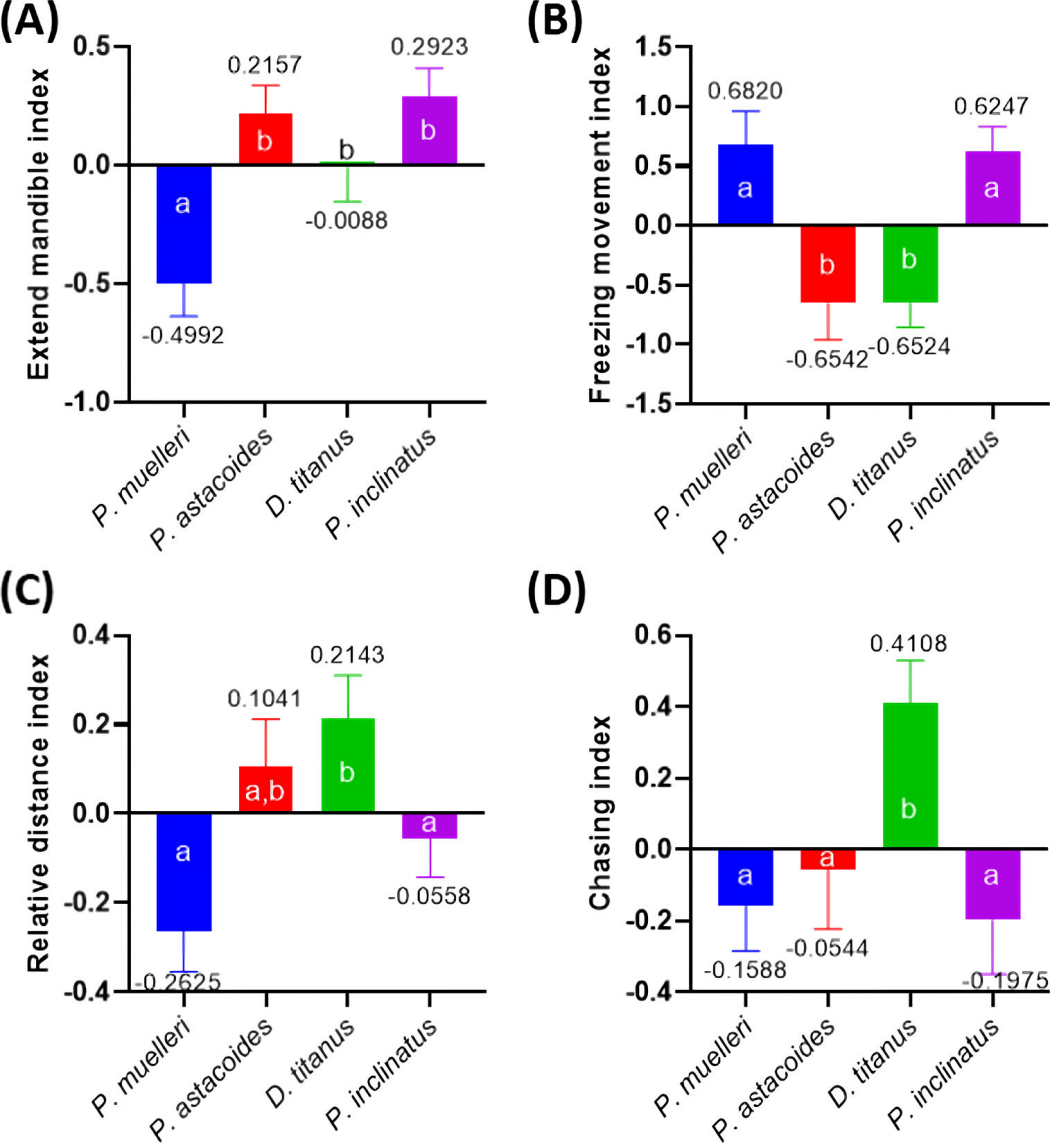
The comparison of several important behavioural endpoint indices that represent aggression activity in inter- species stag beetles. (A) Extended mandible index, (B) freezing movement index, (C) relative distance index and (D) chasing index were measured. The data are displayed as mean ± s.e.m. and statistically analysed by Kruskal–Wallis with multiple comparisons of uncorrected Dunn’s test (*n* = 21).

The analysis of behavioural endpoints for inter-species aggression activity in stag beetles revealed significant differences across four key behaviours: extended mandible, freezing movement, relative distance and chasing interaction. These endpoints were selected based on their consistent differentiation between species, as shown in figures 10 and 11. Figure 13 displays the calculated indices for these behaviours across inter-species pairs, where the indices were determined by subtracting the endpoint values between species in each pair and normalizing them by the total behaviour value. Notable findings include significant differences in the behavioural indices between species, marked by distinct groupings indicated by letters (e.g. ‘a’ and ‘b’) in the figure. The behaviour index of four different species was measured: *P. muelleri* (A), *P. astacoides* (B), *D. titanus* (C) and *P. inclinatus* (D). In the extended mandible endpoint, it showed a higher index value in species B and D, indicating an enhanced aggression pattern compared to other species combinations. Meanwhile, species A showed significantly lower index compared to the other three species (figure 13A). In freezing movement, species with lower aggression levels exhibited higher freezing movement indices as displayed by species A and D (figure 13B), suggesting an inverse relationship between freezing and active aggression. In the relative movement distance, differences in the proximity of individuals during interactions were pronounced, with higher distances correlating with more aggressive pairs. Species C has the highest distance index, followed by species B (figure 13C). Meanwhile, species A and D displayed negative index when compared to other species. Higher chasing interaction indices were observed in species C, aligning with their known behavioural characteristics of territorial aggression (figure 13D). These results underscore the importance of these behavioural endpoints in quantifying aggression across stag beetle species and demonstrate their utility in developing standardized aggression indices for comparative studies.

## 4. Discussion

With the development of new or improved technologies, entomologists now have access to addressing previously obstinate matters, especially in the area of insect behaviour [10]. Our study presents the fundamental application of computer vision for related research fields, such as: image capture, data extraction, and data analysis. Throughout this article, we describe the method for video recording, tracking, and behaviour analyses in stag beetles. Insects are increasingly being studied by measuring their behaviour, and we anticipate that computer vision and deep learning will be increasingly used to measure behaviour, and that these tools and approaches will provide new insights into insect behaviour and answer questions in entomology [22,23].

In this study, we conducted statistical analyses to compare the social behaviour of four stag beetle species, focusing on both intra- and inter-species differences. Our findings revealed significant variations in social interaction and agonistic behaviour among the species. The intra-species comparisons showed consistent patterns of behaviour within each species, while the inter-species comparisons highlighted distinct differences in social dynamics. These results underscore the importance of species-specific behaviours in shaping social interactions among stag beetles. However, the four stag beetle species, *P. muelleri*, *P. astacoides*, *D. titanus* and *P. inclinatus*, do not naturally share the same ecological niche. For example, *P. muelleri* is native to tropical rainforests in northern Queensland, Australia and New Guinea [17,24]. Meanwhile, *P. astacoides*, *D. titanus* and *P. inclinatus* are more commonly found in temperate regions across Asia [25]. Their different habitats suggest that inter-species interactions observed in laboratory conditions may not directly reflect natural encounters. However, studying these interactions can still provide valuable insights into species-specific behavioural traits and evolutionary strategies.

The controlled experimental set-up simplifies complex environmental factors, allowing researchers to focus on species-specific traits. While natural habitats provide dynamic conditions that shape behaviours, these laboratory conditions facilitate standardized comparisons across species. The observed aggression and locomotor activity likely reflect intrinsic behavioural tendencies rather than mere environmental influences. Studying inter-species interactions, even in species that may not naturally encounter each other, offers insights into broader patterns of social behaviour and ecological roles. The findings suggest evolutionary drivers that may influence these interactions, providing a framework for understanding competition, resource allocation and behavioural ecology in other stag beetle species and insects in general.

We also identified potential correlations between behavioural traits and morphological characteristics, such as mandible size and body weight. Larger mandibles were generally associated with increased agonistic behaviour, suggesting that mandible size plays a crucial role in competitive interactions [26,27]. Additionally, body weight was found to influence social interaction time, with heavier individuals engaging in longer interactions [28,29]. These correlations provide valuable insights into the relationship between morphology and behaviour in stag beetles. The metrics of mandible contact and extended mandible ratios were selected based on their established role in aggression displays across stag beetle species. Mandibles serve as critical tools for both offensive and defensive interactions, and their usage is commonly observed in competitive encounters among stag beetles [4,27]. These behaviours are particularly relevant for studying aggression, as they reflect both active engagement and intimidation tactics [29]. Mandible extension ratios play a critical role in stag beetle displays, yet their significance may vary across Lucanidae species depending on ecological pressures and mandible morphology. A study by Emlen [1] underscores the importance of mandible size and form in stag beetle fights, linking exaggerated mandibles to dominance and resource acquisition [1]. Extending this analysis across a broader range of beetle species would test whether mandible extension consistently predicts aggression levels. Such comparative studies could reveal whether behavioural strategies observed here are universally shared or species-specific adaptations.

Based on the established method, we have successfully analysed the behaviour in four different stag beetle species. The rainbow stag beetle (*P. muelleri*) is known for its impressive mandibles, which males use in competitive interactions to secure mating rights. While generally regarded as peaceful, males can exhibit aggressive behaviour during confrontations with rivals [30]. Meanwhile, for *P. astacoides*, this species is known for its sharp mandibles used in fighting [31]. Males exhibit aggressive behaviour, shaking their antennae when angry. The aggressiveness of males and females of *P. inclinatus*, a related species, is mediated by different biogenic amines [32]. Male stag beetles, including *D. titanus*, use their oversized mandibles in battles for females. Their fighting behaviour is essential to understanding the evolution and morphology of their weaponry [4]. Mandible contact, often used in direct fights over resources such as mates or territory, is a common behaviour in male stag beetles. The higher frequency of this behaviour in *D. titanus* and *P. inclinatus* may indicate their strong territoriality and competitive nature. The observed higher levels of chasing and mandible contact behaviours in *D. titanus* and *P. inclinatus* suggest that these species may adopt more aggressive strategies for securing resources or maintaining territories. This aligns with the hypothesis that increased aggression is an adaptation to environments where competition for limited resources is intense [33]. A study on stag beetle battle behaviour explores how anatomical adaptations, such as mandible size, are linked to aggressive interactions and dominance [4]. This provides further evidence for the role of aggression in evolutionary strategies. In contrast, the lower levels of these behaviours in *P. muelleri* and *P. astacoides* may reflect a more passive or defensive approach to interactions.

The jaw morphology and fighting forces in stag beetles have been studied to understand how these anatomical adaptations support their aggressive behaviour [34]. Aggressiveness in stag beetles is influenced by several factors, including biogenic amines and morphological adaptations. It reported that the aggressiveness of males and females of *P. inclinatus* is mediated by different biogenic amines. In males, dopamine increases aggressiveness, while in females, octopamine plays a similar role. These amines act as chemical messengers in the brain, influencing the beetles’ behaviour [32]. Biogenic amines are known to modulate aggression in insects, as evidenced by Stevenson & Rillich [35], who demonstrated their influence on behavioural responses in arthropods. Manipulating mandible extension ratios through targeted treatments or environmental stimuli could reveal causative links between this simple display and aggressive intent [35]. To capture the complexity of ethological behaviours like aggression, multivariate analyses and machine learning models could be applied to behavioural endpoints. By combining mandible extension ratios with locomotion metrics (e.g. freezing, chasing, interaction time), these methods could identify more robust predictors of aggression. Studies such as Yamane *et al*. [36] demonstrated the utility of combining morphological traits with behavioural data to predict insect combat outcomes, offering a potential framework for future analyses [36]. These approaches would enhance the validity of the correlation between mandible extension ratios and aggression by providing broader comparative data, experimental manipulations and integrative analyses [37].

The implications of our findings extend to the broader understanding of the ecology, evolution and conservation of stag beetles. The observed behavioural differences among species can inform ecological studies on habitat preferences and resource competition [38]. From an evolutionary perspective, our results contribute to the understanding of the diversification of social behaviour in stag beetles, shedding light on the adaptive significance of these behaviours [39,40]. Furthermore, the insights gained from this study can aid in the development of conservation strategies to protect stag beetle populations, particularly those that are threatened by habitat loss and environmental changes [41,42]. Based on the current data, we have successfully gained insight into stag beetle behaviours by using the developed method. Our results show prominent results that certain species, such as *D. titanus*, displayed higher aggression activity compared to other species. We hope that the study presented here serves as a starting point for other researchers to delve deeper into how computer vision and deep learning can be applied to their research.

While this study provides meaningful insights into the behavioural ecology of stag beetles and establishes DLC as a valuable tool for analysing complex social interactions, several limitations must be acknowledged. The experimental set-up included a small acrylic plate to facilitate controlled conditions with short observation periods of beetle interactions. Although this enabled precise behavioural recordings, such spatial constraints may have introduced artificial behaviours unrepresentative of natural contexts. For example, confined environments might limit locomotion or intensify aggression levels, as stag beetles typically interact in larger, more dynamic ecological spaces. These conditions should be considered when interpreting the findings. Future research could incorporate larger, habitat-simulating set-ups to validate these results and better replicate natural interaction dynamics. Another concern is the same individuals and only male insects participated in multiple trials to ensure sufficient behavioural data were collected. This approach, while practical, introduces the possibility of bias stemming from prior experiences, such as habituation to the set-up or altered responses due to repeated exposure. To minimize such effects, trials were randomized, and sufficient rest periods were provided between experiments. Nonetheless, this remains a potential limitation. Expanding the sample size, different sexes and developmental stages by including a greater aspects of unique individuals in future studies would enhance the robustness of the conclusions and further mitigate this bias. We agree that a broader range of ecological, physiological and contextual variables may contribute to aggressive behaviours beyond what is currently quantified. In addition, a larger and more diverse training dataset could improve the model’s robustness and generalizability. A broader dataset could potentially enhance the model’s ability to track body parts across different poses and lighting conditions. While our study primarily focuses on body part tracking to define behavioural parameters, future research could incorporate factors such as hormonal influences, environmental stimuli and social hierarchies to provide a more comprehensive picture. By addressing these limitations, future studies can refine the methodology, providing even greater ecological relevance and accuracy in behavioural analyses. Despite these constraints, the findings of this study contribute significantly to the understanding of species-specific behavioural strategies and pave the way for scalable applications of quantitative tools in behavioural ecology.

## Data Availability

The data and raw behavioural data that support the findings of this study are available at Zenodo [43]. The software (DeepLabCut™) that supports the findings of this study is openly available at GitHub (https://github.com/deeplabcut). Electronic supplementary material is available online [44].
